# Prevalence and Risk Factors of Hypernatremic Dehydration in Exclusively Breastfed Neonates: A Systematic Review and Meta-Analysis

**DOI:** 10.3390/jcm15134975

**Published:** 2026-06-26

**Authors:** María José Maldonado, Eduardo Tuta-Quintero, Isabella Criado Quintero, Andrea V. Zambrano, Maria F. Velazco, Sergio Agudelo-Perez

**Affiliations:** 1 Department Pediatrics, School of Medicine, Universidad de La Sabana, Chía 250001, Colombia; maria.maldonado5@unisabana.edu.co; 2 Department of Epidemiology, School of Medicine, Universidad de La Sabana, Chía 250001, Colombia; eduardotuqu@unisabana.edu.co; 3 School of Medicine, Universidad de La Sabana, Chía 250001, Colombia; isabellacrqu@unisabana.edu.co (I.C.Q.); andreazalu@unisabana.edu.co (A.V.Z.); mariavebe@unisabana.edu.co (M.F.V.); 4 Doctoral Programme in Clinical Sciences, School of Medicine, Universidad de La Sabana, Campus del Puente del Común, Km. 7, Autopista Norte de Bogotá, Chía 250001, Colombia

**Keywords:** neonatal hypernatremic dehydration, exclusive breastfeeding, newborn, hypernatremia, neonatal dehydration, prevalence, risk factors, systematic review, meta-analysis

## Abstract

**Background/Objectives**: Exclusive breastfeeding improves infant health and development, while suboptimal breastfeeding increases risks of hyperbilirubinemia and neonatal hypernatremic dehydration (NHD). This study aims to estimate the prevalence of NHD associated with exclusive breastfeeding and to identify maternal and neonatal risk factors through a systematic review and meta-analysis. **Methods**: This systematic review followed PRISMA 2020 guidelines. A comprehensive search was conducted in PubMed, Scopus, Web of Science, LILACS, and the Cochrane Central Register of Controlled Trials from February to March 2025 without language or publication year restrictions. Observational studies evaluating healthy term neonates exclusively breastfed and diagnosed with NHD within the first 28 days of life were included. Two independent meta-analyses were performed to estimate pooled prevalence and associated risk factors using random-effects models. Methodological quality was assessed using the Newcastle–Ottawa Scale (NOS) and the Joanna Briggs Institute (JBI) tool. **Results**: A total of 97 studies were identified, and 13 met the inclusion criteria. Ten studies were included in the prevalence meta-analysis and seven in the risk factor meta-analysis. The pooled prevalence of NHD was 1.4% (95% CI: 0.0–5.0%), with high heterogeneity (I^2^ = 99.6%). Delayed initiation of breastfeeding (OR 6.02; 95% CI: 2.85–12.73), excessive neonatal weight loss > 10% (OR 69.28; 95% CI: 0.00–1.87 × 10^12^), low urine output (OR 8.51; 95% CI: 2.86–25.29), and maternal primiparity (OR 3.66; 95% CI: 1.67–8.02) were identified as the main risk factors. Poor breastfeeding technique, inadequate latch, and lack of early postnatal follow-up were consistently associated with NHD. **Conclusions**: NHD is a clinically relevant condition among exclusively breastfed term newborns. Early breastfeeding assessment, monitoring of neonatal weight loss and hydration status, and strengthened maternal support are essential to prevent severe complications.

## 1. Introduction

Exclusive breastfeeding during the first six months of life promotes optimal growth and health outcomes [1,2]. It is associated with multiple short-, medium-, and long-term benefits, including reduced infant mortality, lower incidence of infections, particularly respiratory tract infections, and fewer episodes of diarrhea [3]. Furthermore, it has been associated with improved neurodevelopment, lower risk of obesity, higher intelligence quotient, and protective effects against behavioral disorders and executive function impairments later in life [3,4]. In contrast, suboptimal breastfeeding in low-risk neonates is associated with an increased incidence of hyperbilirubinemia and neonatal hypernatremic dehydration (NHD) [5,6].

NHD is commonly defined by elevated serum sodium levels, excessive neonatal weight loss, and clinical signs of dehydration during the first postnatal days. Reported sodium thresholds vary across studies, most commonly ranging from >145 to ≥150 mEq/L [7,8,9,10,11]. The estimated prevalence ranges from 1.6% to 6.5% [7,8,9,10,11], and the condition is closely associated with insufficient milk intake during unsuccessful breastfeeding establishment, particularly among exclusively breastfed neonates [5].

Importantly, neonatal hypernatremic dehydration should not be interpreted as a complication of breastfeeding. Exclusive breastfeeding remains the optimal nutritional strategy for healthy term neonates and provides substantial short- and long-term benefits for both infants and mothers. NHD is generally considered a preventable condition related to insufficient milk intake during unsuccessful breastfeeding establishment and delayed recognition of feeding difficulties during the early postnatal period.

Although several etiologies have been proposed, unsuccessful breastfeeding establishment and insufficient milk transfer are currently recognized as the main underlying mechanisms associated with NHD [5,12,13], making it a frequent complication of feeding difficulties during the first week of life [14]. Inadequate milk intake during the early postnatal period may result from ineffective latch, delayed lactogenesis, infrequent feeding, or other breastfeeding-related difficulties, leading to dehydration and hypernatremia [9]. Clinically, NHD may present with fever, excessive weight loss, jaundice, lethargy, decreased urine output, and decreased stool frequency [5,9].

NHD also contributes to early weaning, negatively affecting exclusive breastfeeding rates and potentially leading to severe complications, including neurological, renal, and electrolyte disturbances, and even death [15,16]. Interventions aimed at improving breastfeeding techniques have been shown to reduce excessive weight loss, prolong exclusive breastfeeding during the first six months, and improve nutritional outcomes during the first year of life [17]. However, early detection remains limited owing to nonspecific clinical manifestations and the lack of effective screening strategies [10]. Therefore, this systematic review and meta-analysis aimed to estimate the prevalence of neonatal hypernatremic dehydration among exclusively breastfed newborns and to identify associated maternal and neonatal risk factors. Additionally, this review sought to explore the methodological and clinical heterogeneity across studies and provide evidence that may contribute to improving early breastfeeding monitoring and follow-up strategies aimed at preventing severe neonatal complications.

## 2. Methods

This systematic review and meta-analysis were conducted and reported according to the Preferred Reporting Items for Systematic Reviews and Meta-Analyses (PRISMA) 2020 guidelines. The completed PRISMA checklist is provided in the Supplementary Material (Supplementary Table S1).

### 2.1. Search Strategy and Study Identification

A comprehensive literature search was conducted between February and March 2025 in the electronic databases PubMed, Scopus, Web of Science (WoS), LILACS, and the Cochrane Central Register of Controlled Trials. In addition, a secondary search was performed using the snowballing method by reviewing the reference lists of the included studies to identify potentially relevant articles that were not detected in the initial search. No language or publication year restrictions were imposed.

The search strategy combined indexed terms (MeSH) and keywords related to the population, exposure, and outcomes of interest of the study. The terms included neonates, newborns, breastfeeding, exclusive breastfeeding, hypernatremia, neonatal hypernatremic dehydration, prevalence, and risk factors. For PubMed, the following search equation was used: (((Infant, Newborn) AND ((Breast Feeding) OR (Breastfeeding, Exclusive))) AND (((Hypernatremia) OR (Neonatal Hypernatremic Dehydration)) OR (Hypernatremic Dehydration))) AND ((Prevalence) OR (Risk Factors)). Equivalent adaptations of this strategy were applied to the other databases. The full details of the database-specific search strategies are presented in Supplementary Table S2.

### 2.2. Eligibility Criteria

Studies including term neonates (≥37 weeks of gestational age) exclusively breastfed from birth and evaluated for neonatal hypernatremic dehydration during the first 28 days of life were included. Studies were required to include neonates without major congenital anomalies, severe comorbidities, or conditions requiring prior Neonatal Intensive Care Unit (NICU) admission before the episode of neonatal hypernatremic dehydration. Observational studies, including cross-sectional, cohort, and case–control designs, that reported sufficient data to estimate the prevalence and/or evaluate maternal or neonatal risk factors associated with NHD were included.

Systematic reviews, narrative reviews, case reports, case series, conference abstracts, secondary studies, and studies with insufficient information for data extraction were excluded.

### 2.3. Operational Definitions

NHD was defined as serum sodium levels ≥ 145 mEq/L, generally occurring within the first seven days of life, and associated with clinical signs of dehydration, such as jaundice, decreased intake, excessive weight loss, oliguria, and neurological manifestations (irritability or lethargy).

### 2.4. Risk Factors Considered

All variables reported to be potentially associated with an increased risk of developing NHD were included. The main factors analyzed comprised social variables, such as maternal educational level, social support, and pregnancy planning or desire; biological factors, including maternal age and parity; and clinical factors, such as the mode of delivery, perinatal conditions, and neonatal clinical signs.

### 2.5. Study Selection and Inclusion

Study selection was conducted independently and in a blinded manner by four investigators (SA, IC, AZ, and MV). During the initial screening phase, titles and abstracts were independently reviewed using the Rayyan^®^ platform after the removal of duplicate records. Potentially eligible articles were retrieved in full text and independently assessed by each reviewer. Discrepancies at any stage were resolved through discussion and consensus agreement.

### 2.6. Data Extraction and Synthesis

Data extraction was performed independently and in duplicate by the reviewers using a standardized spreadsheet in Microsoft Excel. The following information was collected from each study: (a) first author, publication year, and country; (b) study design; (c) population characteristics and total number of included patients; (d) age at hospital admission; (e) diagnostic criteria used to define NHD; (f) percentage of weight loss at admission relative to birth weight; (g) serum sodium levels at admission; and (h) relevant conclusions or main findings.

For the prevalence analysis, the following data were extracted: (a) total number of evaluated patients and (b) number of patients with NHD.

For the risk factor analysis, the following data were collected whenever available: (a) number of patients with NHD exposed to the risk factor, (b) number of patients without NHD exposed to the risk factor, (c) number of patients with NHD without exposure, (d) number of patients without NHD without exposure, and (e) odds ratios (ORs) and their 95% confidence intervals (95% CIs). Data were organized and synthesized according to the study objectives, distinguishing information intended for prevalence estimation from that used for risk factor analyses.

### 2.7. Methodological Quality Assessment and Risk of Bias

The methodological quality of cohort and case–control studies was assessed using the Newcastle–Ottawa Scale (NOS), which classifies the risk of bias as low, moderate, or high according to the obtained score. The Joanna Briggs Institute (JBI) critical appraisal tool was used for cross-sectional studies. All assessments were performed independently by the investigators, and disagreements were resolved through consensus.

### 2.8. Statistical Analysis

Two independent meta-analyses were performed: one to estimate the pooled prevalence of NHD and the other to evaluate the associated risk factors.

### 2.9. Prevalence Meta-Analysis

The pooled prevalence of NHD was estimated from the proportions reported in the included studies, calculating 95% confidence intervals (95% CIs) using the exact Clopper–Pearson method. Proportions were pooled using a random-effects model with logit transformation (PLOGIT), employing the maximum likelihood (ML) estimator for between-study variance (τ^2^). Heterogeneity was assessed using Cochran’s Q statistic, τ^2^, and I^2^, considering I^2^ values above 75% to be indicative of substantial heterogeneity. Results were expressed as pooled prevalence with 95% CIs and back-transformed to the original scale using inverse logit transformation.

Publication bias was explored using funnel plot inspection and Egger’s test based on a mixed-effects meta-regression model. A *p*-value < 0.05 was considered indicative of asymmetry, although the interpretation was performed cautiously, given the high observed heterogeneity, which could reflect clinical or methodological variability in addition to publication bias.

Additional subgroup analyses were performed according to the serum sodium threshold used to define hypernatremia (≥145 vs. ≥150 mEq/L) and study setting (population-based vs. hospital/NICU-based studies) to further explore potential sources of heterogeneity. A sensitivity analysis excluding studies using non-standard case definitions, including studies primarily evaluating excessive neonatal weight loss rather than biochemically confirmed neonatal hypernatremic dehydration, was conducted.

Analyses were conducted in R (version 4.5.0) using the meta package (version 8.2.0) and metaprop function.

### 2.10. Risk Factor Meta-Analysis

A random-effects meta-analysis was performed to assess the risk factors associated with NHD. The logarithm of the odds ratio (logOR) and its standard error (SE) were calculated from the reported 95% CIs for each study. Between-study variance (τ^2^) was estimated using the DerSimonian–Laird (DL) method, and the Hartung–Knapp–Sidik–Jonkman (HK) adjustment was applied to obtain more robust confidence intervals.

Heterogeneity was assessed using Cochran’s Q statistic and I^2^, considering *p* < 0.10 to be statistically significant. Results were expressed as odds ratios (ORs) with 95% CIs, obtained by exponentiating the pooled logORs. Only risk factors reported in at least two studies were included in the meta-analysis. Factors analyzed in a single study were described qualitatively to ensure transparency and provide complementary evidence for the findings. Publication bias was not assessed in this analysis because most risk factors were investigated in fewer than ten studies, a condition that limits the validity of asymmetry tests. Analyses were conducted in R (version 4.5.0) using the meta (version 8.2.0) and metafor (version 4.8.0) packages.

## 3. Results

**Figure 1:**
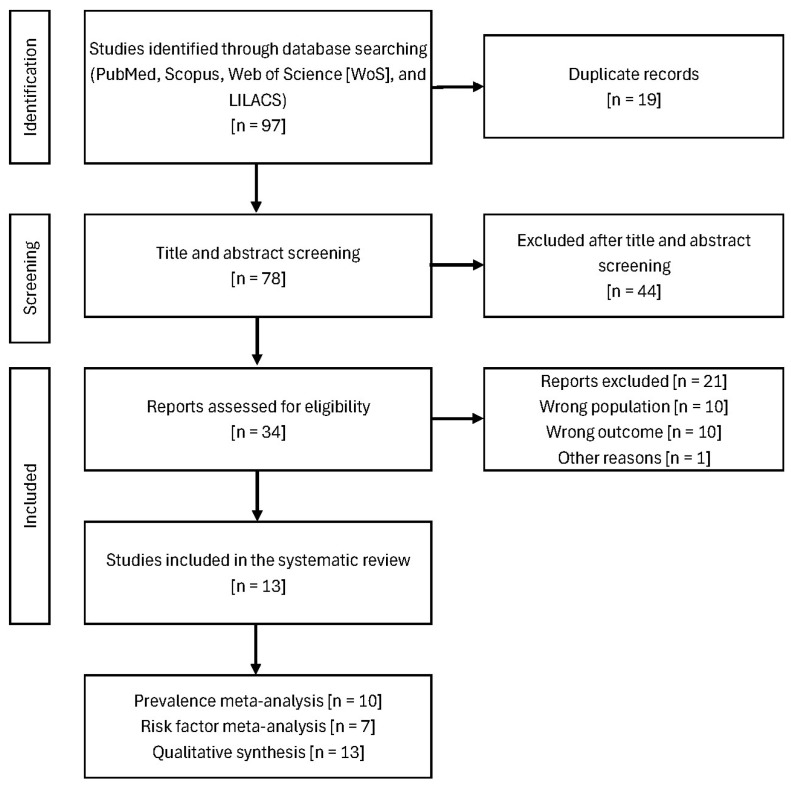
PRISMA flow diagram.

A total of 97 studies were included. After removing 19 duplicates, 78 studies were screened based on their titles and abstracts, of which 44 were excluded. Thirty-four full-text articles were assessed for eligibility, and 21 were excluded based on methodological or population-related criteria. Finally, 13 studies met the inclusion criteria: 10 were included in the prevalence meta-analysis, seven in the risk factor meta-analysis, and all 13 in the qualitative synthesis (Figure 1).

### 3.1. Included Studies, General Characteristics, and Definition of NHD

The included studies were conducted in university or tertiary care hospitals across several countries, predominantly Turkey (Table 1). Most of them were public hospitals with specialized neonatal units. The study population mainly included first-born male neonates delivered by caesarean section. Ten studies evaluated prevalence, and seven analyzed risk factors related to breastfeeding and maternal characteristics.

Across the included studies, the mean age at hospital admission was approximately 5 days (range, 1–16 days). The mean weight loss among neonates with NHD was approximately 9% (range: 1.3–27%). Serum sodium levels at admission generally ranged from 150 to 190 mEq/L. Considerable heterogeneity was observed in the diagnostic criteria used; some studies defined hypernatremia as serum sodium ≥ 145 mEq/L, whereas others used higher thresholds. Several studies have also incorporated clinical criteria, such as signs of dehydration, excessive weight loss, jaundice, oliguria, and lethargy. All studies included healthy term neonates who were exclusively breastfed and hospitalized within the first 28 days of life.

### 3.2. Quality Assessment and Risk of Bias

The methodological quality assessment using the NOS for cohort studies (Supplementary Table S3) showed scores ranging from 7 to 9 stars. Bolat et al. (2013) achieved 9/9 stars, whereas Ünver Korgalı et al. (2017) and Miyoshi et al. (2020) achieved 8/9 stars. Arora et al. (2024) and Kenaley Greenspan et al. (2020) obtained 7/9 stars, with limitations in comparability due to the absence of multivariate adjustment [6,9,18,22].

Among the case–control studies assessed with the NOS (Supplementary Table S4), Boskabadi et al. (2010) achieved 9/9 stars; Ozbek et al. (2008) and Celik et al. (2021) achieved 8/9; and Çağlar et al. (2006) achieved 7/9, with limitations related to control selection, comparability, and non-response rates [5,20,26,27]. Overall, the item “same non-response rate in both groups” was frequently rated as “Unclear.”

Cross-sectional studies evaluated using the Joanna Briggs Institute (JBI) tool (Supplementary Table S5) generally demonstrated good methodological quality. However, confounding factor management was marked as “Not applicable” in all studies, and in Carvalho et al. [25], the clarity of inclusion criteria was rated as “Unclear,” resulting in an adjusted score of 5/6 applicable criteria. Overall, the global quality of the included studies was adequate.

### 3.3. Qualitative Synthesis

Poor breastfeeding technique has been consistently identified as an important risk factor for neonatal hypernatremic dehydration [6,9,19,21,23]. One study reported that 73.5% of infants had low LATCH scores [6]. Problems such as poor latch, ineffective sucking, infrequent feeding sessions, and lack of postnatal follow-up are associated with an increased risk [9,19,21,23]. Carvalho et al. reported that hospital admissions for all dehydration episodes occurred between the second and fifth days of life; furthermore, no timely weight monitoring or professional home support was documented during this period [25]. Therefore, the authors recommend early evaluation within the first 15 days of life, ideally 48–72 h after hospital discharge, to identify warning signs of insufficient or failed breastfeeding that may predispose neonates to dehydration.

Among newborns with weight loss ≥ 10%, serum sodium levels ≥ 150 mEq/L were considered criteria for admission to the neonatal intensive care unit because of their association with severe neurological complications such as cerebral venous thrombosis, pontine myelinolysis, cerebral edema, or seizures [22]. In addition, a positive and statistically significant correlation was observed between the percentage of weight loss and serum sodium concentration (r = 0.67; *p* < 0.01). The main reasons for hospital admission among infants with NHD were fever (50%), lethargy (45.3%), and jaundice (39.6%), together with excessive weight loss [5,19,21]. Bilgin et al. reported that jaundice and fever were present in 64.4% and 56.4% of cases, respectively, both being frequent presenting signs of this condition [19]. The authors suggested that fever may represent an additional risk indicator in neonates with excessive weight loss and that hypernatremia may be associated with significant hyperbilirubinemia, potentially increasing the risk of bilirubin encephalopathy due to blood–brain barrier disruption.

In a study including 159 term infants hospitalized for NHD associated with exclusive breastfeeding, male sex predominated, accounting for 64.2% of the cases [9]. Carvalho et al. found that 33% of mothers in the study group had primary hypogalactia, and 17% presented with psychiatric disorders, although these conditions were not further specified [25]. Ozbek et al. identified previous maternal psychiatric morbidity as a significant risk factor, with a prevalence of 57.1% among affected mothers versus 18.6% in controls (*p* = 0.003), although no specific diagnosis was reported. Additionally, a poor maternal relationship with the mother’s own mother was significantly associated with the neonatal hypernatremic dehydration group, with a prevalence of 36.8% compared to 9.6% in the control group (*p* = 0.026). Similarly, unplanned pregnancies were associated with an increased risk (52.4% vs. 20.9%; *p* = 0.020). Finally, maternal self-perception as an inadequate or uncertain mother was significantly more frequent in the affected group (28.6% vs. 4.6%; *p* = 0.012), with a crude OR of 8.2 (95% CI: 1.489–45.161) [26].

The study by Çağlar et al., which included 18 neonates with weight loss ≥ 10% and 72 controls, found no significant differences in the maternal educational level between the groups. However, other maternal factors, such as primiparity and technical breastfeeding difficulties (delayed initiation of the first feeding, poor latch, and breast abnormalities), appear to play a more relevant role in neonatal clinical outcomes [20].

### 3.4. Quantitative Synthesis

**Figure 2:**
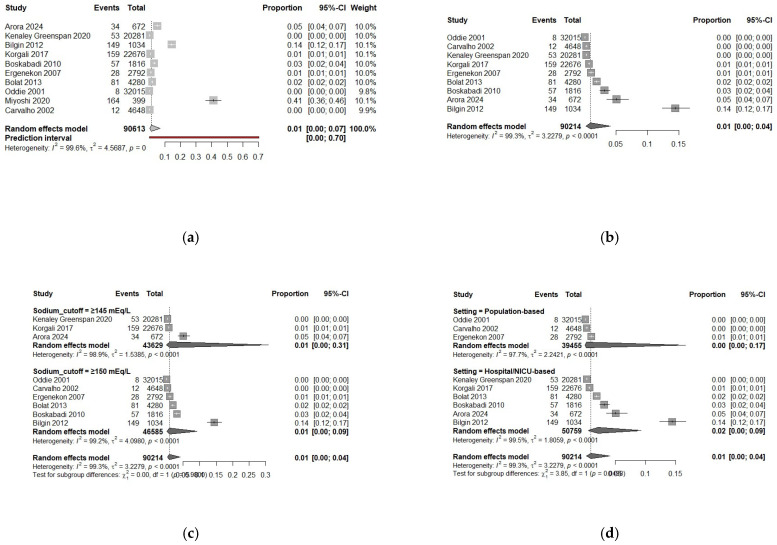
Forest plots of the meta-analysis and sensitivity analyses for the prevalence of neonatal hypernatremic dehydration in exclusively breastfed term neonates. (**a**) Main meta-analysis including all ten studies; (**b**) sensitivity analysis excluding the study by Miyoshi et al. due to methodological differences in case definition, primarily based on excessive neonatal weight loss rather than biochemically confirmed neonatal hypernatremic dehydration; (**c**) subgroup analysis according to serum sodium cutoff used to define hypernatremia (≥145 vs. ≥150 mEq/L); and (**d**) subgroup analysis according to study setting (population-based vs. hospital/NICU-based studies).

The individual prevalence estimates of NHD varied widely, ranging from 0.0% to 41.0%, reflecting marked differences across studies. The meta-analysis showed a pooled prevalence of 1.4% (95% CI: 0.0–5.0%) (Figure 2a). Between-study heterogeneity was extremely high (I^2^ = 99.6%, τ^2^ = 4.63, *p* < 0.001).

Sensitivity analyses excluding the study by Miyoshi et al., which primarily defined cases based on excessive neonatal weight loss, yielded prevalence estimates comparable to those of the primary analysis, with persistent substantial heterogeneity (Figure 2b).

Subgroup analyses according to the serum sodium threshold (≥145 vs. ≥150 mEq/L) showed similar pooled prevalence estimates between subgroups, while heterogeneity remained extremely high in both analyses (Figure 2c), indicating that sodium cutoff differences alone did not fully explain the observed between-study variability.

Additional subgroup analyses according to the study setting demonstrated higher pooled prevalence estimates in hospital/NICU-based studies than in population-based cohorts (Figure 2d). Although the study setting contributed significantly to between-study heterogeneity, substantial heterogeneity persisted within both subgroups, suggesting that additional methodological and clinical differences across studies also influenced prevalence estimates.

**Figure 3:**
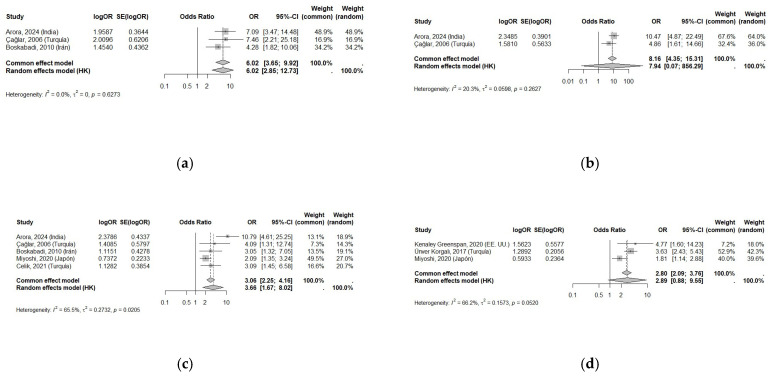
Forest plots of the meta-analysis of breastfeeding-related and maternal/perinatal factors associated with neonatal hypernatremic dehydration. Notes: (**a**) delayed initiation of breastfeeding; (**b**) low breastfeeding frequency; (**c**) maternal primiparity; and (**d**) cesarean delivery.

The meta-analysis of breastfeeding-related and maternal/perinatal factors associated with neonatal hypernatremic dehydration is presented in Figure 3. Delayed initiation of breastfeeding was associated with a pooled OR of 6.02 (95% CI: 2.85–12.73; I^2^ = 0.0%; *p* < 0.001) (Figure 3a). Low breastfeeding frequency showed a pooled OR of 7.94 (95% CI: 0.07–856.29; I^2^ = 20.3%; *p* = 0.263) (Figure 3b). Maternal primiparity was associated with a pooled OR of 3.66 (95% CI: 1.67–8.02; I^2^ = 65.5%; τ^2^ = 0.2732; *p* = 0.021) (Figure 3c). Cesarean delivery showed a pooled OR of 2.89 (95% CI: 0.88–9.55; I^2^ = 66.2%; τ^2^ = 0.1573; *p* = 0.052) (Figure 3d).

**Figure 4:**
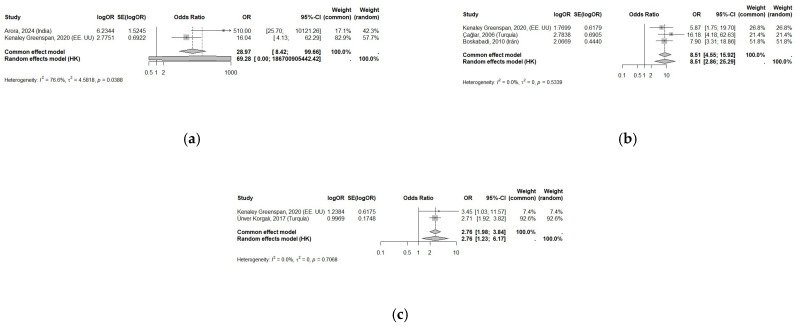
Forest plots of the meta-analysis of neonatal clinical markers and characteristics associated with neonatal hypernatremic dehydration. Notes: (**a**) excessive neonatal weight loss (>10% of birth weight); (**b**) low urinary output; and (**c**) gestational age greater than 40 weeks.

The meta-analysis of neonatal clinical markers and characteristics associated with neonatal hypernatremic dehydration is presented in Figure 4. Excessive neonatal weight loss (>10% of birth weight) showed a pooled OR of 69.28 (95% CI: 0.00–1.87 × 10^12^; I^2^ = 76.6%; *p* = 0.039) (Figure 4a). However, the estimate demonstrated substantial imprecision, with a markedly wide confidence interval, reflecting considerable statistical instability in the included studies. Low urine output (<6 voids per day) was associated with a pooled OR of 8.51 (95% CI: 2.86–25.29; I^2^ = 0%; τ^2^ = 0; *p* = 0.534) (Figure 4b). Finally, gestational age > 40 weeks showed a pooled OR of 2.76 (95% CI: 1.23–6.17; I^2^ = 0%; τ^2^ = 0; *p* = 0.707) (Figure 4c).

### 3.5. Publication Bias

**Figure 5:**
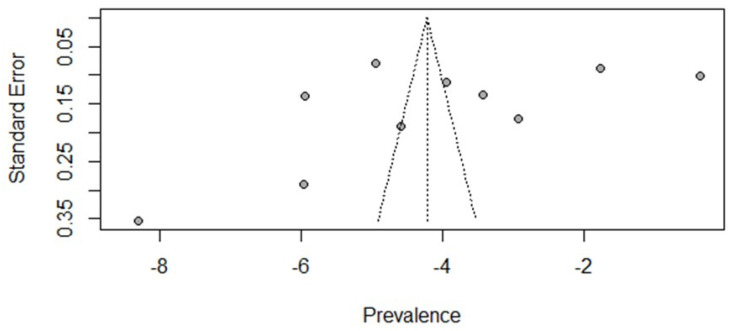
Funnel plot for the assessment of publication bias in the prevalence meta-analysis.

The funnel plot showed an asymmetric distribution, with several studies located away from the effect line, particularly those with small sample sizes and extreme prevalence estimates (Figure 5), suggesting publication bias. Egger’s test suggested funnel plot asymmetry (z = −2.93; *p* = 0.003). However, this finding should be interpreted cautiously, given the limited number of included studies and the extremely high between-study heterogeneity, which may substantially affect the reliability of the publication bias assessment in prevalence meta-analyses.

## 4. Discussion

This study conducted a systematic review and meta-analysis to estimate the prevalence and risk factors associated with NHD in exclusively breastfed newborns. The results showed an overall NHD prevalence of 1.4%, and the main identified risk factors were delayed initiation of breastfeeding, excessive neonatal weight loss, maternal primiparity, and cesarean delivery. These findings highlight the influence of maternal and perinatal factors on the development of this condition and underscore the need to strengthen breastfeeding support and follow-up strategies from the time of birth.

The prevalence of NHD found in this study (1.4%) is comparable to that reported by Zakerihamidi (1.38–6.45%) [10]. However, important differences exist between the analyzed populations. In a review by Zakerihamidi, near-term neonates (≥34 weeks) and low-birth-weight infants were included, whereas our study considered only healthy term neonates with appropriate weight for gestational age, without perinatal complications, and exclusively breastfed from birth. These stricter criteria allow for a more homogeneous estimation of prevalence within a specific population, reducing the heterogeneity observed in previous studies. Although the promotion of exclusive breastfeeding remains a fundamental public health strategy, these findings highlight the need to implement clinical surveillance measures that allow for the early identification of signs of insufficient intake and the prevention of complications such as NHD.

Qualitative synthesis demonstrated a consistent relationship between poor breastfeeding techniques and the development of NHD. Difficulties related to latching, positioning, and sucking were identified as key determinants of ineffective milk transfer. This finding is consistent with previous studies; for example, one prospective study reported a mean LATCH score of 4.29 in NHD cases compared with 8.08 in the control group, demonstrating significant differences in breastfeeding efficacy. In the same study, delayed initiation of breastfeeding and excessive neonatal weight loss were identified as associated factors [6]. These findings emphasize the importance of strengthening education and support for breastfeeding mothers and healthcare personnel, starting in the early postnatal period.

Excessive neonatal weight loss was also strongly associated with NHD in the quantitative synthesis. However, this finding should be interpreted with caution because of the substantial statistical imprecision observed and the extremely wide confidence intervals of the pooled estimates. In addition, several included studies incorporated excessive weight loss into the operational definition of neonatal hypernatremic dehydration, introducing potential diagnostic circularity and limiting the interpretation of weight loss as an independent risk factor. From a clinical perspective, excessive neonatal weight loss may be more appropriately interpreted as an early marker of evolving breastfeeding failure and insufficient milk transfer rather than as an isolated etiological factor [28]. These findings reinforce the importance of close postnatal monitoring of neonatal weight trajectories during the first few days of life, particularly among exclusively breastfed infants.

An additional consideration is the substantial heterogeneity in the assessment of breastfeeding difficulties across the included studies. While some studies used structured breastfeeding assessment tools or predefined clinical criteria, others relied primarily on subjective clinical evaluations or indirect markers of ineffective feeding. Consequently, the term “poor breastfeeding technique” likely encompasses different constructs across studies, limiting the direct comparability between reports [29]. Moreover, inadequate milk transfer during the neonatal period is a multifactorial process that extends beyond latch or positioning difficulties alone and may also involve delayed lactogenesis II, maternal breast pain, ineffective suckling, infrequent feeding opportunities, infant oral dysfunction, and maternal perception of low milk supply [29,30]. Therefore, breastfeeding dysfunction should be interpreted as a complex interaction among maternal, infant, and healthcare-related factors rather than as an isolated technical problem. These considerations may partly explain the heterogeneity observed across studies and underscore the need for more standardized and multidimensional breastfeeding assessment approaches in future research.

Several studies have identified maternal primiparity, cesarean delivery, delayed breastfeeding initiation, low feeding frequency, and breastfeeding difficulties as important factors associated with neonatal hypernatremic dehydration (NHD) [16,19,22]. However, current evidence suggests that NHD should not be interpreted as a consequence of breastfeeding itself but rather as a complication arising from insufficient milk intake during unsuccessful breastfeeding establishment. In this context, ineffective milk transfer may result from a complex interaction of maternal, neonatal, psychosocial, and healthcare-related factors, rather than isolated technical difficulties alone. Recent studies have emphasized that inadequate breastfeeding support, delayed recognition of feeding difficulties, insufficient maternal education, and lack of early postnatal follow-up may substantially contribute to the development of NHD [7,31].

Our meta-analysis identified maternal primiparity as a relevant risk factor for NHD. This association may be partially explained by reduced maternal experience in recognizing early signs of ineffective feeding, difficulties with breastfeeding positioning and latching, lower confidence during breastfeeding establishment, and delayed identification of insufficient neonatal intake. Maternal conditions associated with delayed lactogenesis II, including cesarean delivery, gestational diabetes, hypertension, hypothyroidism, breast disorders, nipple abnormalities, postpartum pain, and maternal fatigue, may interfere with adequate milk production and transfer during the critical first postnatal days [7,16,19]. Recent evidence has also highlighted the importance of healthcare system-related factors, including early hospital discharge, inadequate breastfeeding counseling, and limited access to structured breastfeeding support programmes, which may delay the recognition of evolving dehydration and excessive neonatal weight loss [31].

Maternal psychosocial and emotional factors may also contribute to unsuccessful breastfeeding and impaired milk transfer. Previous studies have associated postpartum depression, anxiety, stress, low maternal self-efficacy, and lack of social support with breastfeeding difficulties and reduced breastfeeding duration [32,33]. Likewise, social vulnerability, maternal insecurity regarding milk supply, poor maternal support networks, and difficulties in adapting to the maternal role may negatively affect mother–infant interaction and breastfeeding effectiveness. These findings reinforce the importance of understanding NHD within a broader biopsychosocial and healthcare framework rather than as an isolated feeding technique problem. In this context, early and structured breastfeeding assessment, close postnatal follow-up, maternal education, and timely lactation support are essential to prevent severe complications such as excessive weight loss, hyperbilirubinemia, and neonatal hypernatremic dehydration [29,31,34].

Importantly, our findings should not be interpreted as evidence against exclusive breastfeeding, which remains the optimal feeding strategy for healthy term neonates. Neonatal hypernatremic dehydration appears to be primarily associated with insufficient milk intake during unsuccessful breastfeeding establishment, frequently related to delayed recognition of ineffective milk transfer, inadequate breastfeeding support, delayed lactogenesis, and insufficient postnatal follow-up. Early and close postnatal follow-up is critical for preventing severe complications related to insufficient milk intake during breastfeeding establishment, particularly among mother–infant dyads at risk of delayed lactogenesis or ineffective milk transfer.

Structured breastfeeding assessment and early identification of feeding difficulties may facilitate timely interventions before the development of neonatal hypernatremic dehydration, significant hyperbilirubinemia, or excessive neonatal weight loss [34]. In clinical practice, neonates with NHD frequently present during the first week of life with jaundice, lethargy, fever, or excessive weight loss rather than overt signs of dehydration, highlighting the importance of early monitoring strategies, timely outpatient follow-up after discharge, and careful assessment of neonatal feeding adequacy and weight trajectories [35]. In this context, the prevention of NHD depends not on limiting breastfeeding promotion but on strengthening early lactation assessment, maternal education, and timely identification of feeding difficulties during the first few postnatal days.

### Limitations

The methodological heterogeneity observed across studies complicates comparisons and limits the early identification of the disease in clinical practice. Standardization of diagnostic criteria would improve early detection, facilitate the comparison of epidemiological data, and optimize clinical interventions aimed at preventing morbidity and mortality associated with NHD in exclusively breastfed neonates.

Finally, an important finding of this review was the high variability in the diagnostic criteria used to define NHD. The included studies employed different serum sodium thresholds (≥145 mEq/L to >150 mEq/L), different neonatal weight loss criteria (generally ≥10%), and variable presence of clinical signs, such as fever or overt dehydration. Some studies relied exclusively on serum sodium levels, whereas others combined biochemical criteria with clinical manifestations.

Overall, the findings of this review confirm that neonatal hypernatremic dehydration is a clinically relevant condition that may affect even healthy term newborns with appropriate birth weights. Identification of risk factors and implementation of early follow-up strategies are essential to prevent this condition and ensure safe and effective breastfeeding.

## 5. Conclusions

NHD has an estimated prevalence of 1.4% in exclusively breastfed newborns, with high heterogeneity across studies. The main identified risk factors were delayed initiation of breastfeeding, weight loss > 10%, low urine output, and maternal primiparity. Evidence also highlights the role of poor breastfeeding techniques and lack of early postnatal follow-up. These findings underscore the importance of early clinical monitoring, systematic breastfeeding assessment, and strengthening maternal support to prevent complications in healthy neonates.

## Figures and Tables

**Table 1:** Characteristics of included studies evaluating neonatal hypernatremic dehydration in exclusively breastfed term newborns.

Study	Design	Population/N	Inclusion Criteria	Exclusion Criteria	Timing of Assessment	Diagnostic Criteria	Weight Loss (NHD)	Weight Loss (Non-NHD)	Reported Sodium Level (Non-NHD)	Reported Sodium Level (NHD)
Arora, 2024, India [6]	Prospective observational study	672 term newborns	GA ≥ 37 weeks, BW ≥ 2500 g, exclusive breastfeeding	Congenital disorders, chronic maternal diseases	Admission day: 3–5 days	Clinical diagnosis, serum sodium ≥ 145 mEq/L and weight loss ≥ 10%	11.4% ± 2.3%	2.8% ± 1.6%	136 ± 2.3 mEq/L	145 mEq/L
Kenaley Greenspan [18]	Retrospective cohort study	20,281 newborns; 75 hospitalized for fever, suspected dehydration, some with hypernatremia.	Newborns exclusively breastfed, born in hospital	Prematurity, infections, underlying medical conditions	Admission day: 1–4 days Median age: 2 days	Fever, weight loss ≥ 10%, signs of dehydration and serum sodium ≥ 145 mEq/L	7.9% ± 2.1%	2.3% ± 1.7%	139 ± 1.8 mEq/L	147 ± 3.7 mEq/L
Bilgin, 2012, Turkey [19]	Retrospective study	149 term newborns hospitalized with dehydration among 1034 hospitalized neonates	GA ≥ 37 weeks, breastfed infants with clinical signs of dehydration	Prematurity, formula feeding, congenital or metabolic diseases	Admission day: 4–12 days Mean age: 7 days	Clinical diagnosis, serum sodium > 150 mEq/L	13% ± 6.3%	NR	NR	Median 155 mEq/L (range 150–190 mEq/L)
Ünver Korgalı, 2017, Turkey [9]	Retrospective study	159 term newborns exclusively breastfed	GA ≥ 37 weeks, BW: 2500–4500 g, exclusive breastfeeding	>28 days of life, congenital anomalies, metabolic diseases, asphyxia, sepsis, intrauterine infections, neurological diseases	Admission day: 1–16 days	Clinical diagnosis, serum sodium ≥ 146 mEq/L	10.6% ± 4.6%	8.1% ± 3.1%	147.5 ± 1.0 mEq/L	153.8 ± 5.6 mEq/L
Çağlar, 2006, Turkey [20]	Nested case–control study	90 term newborns (18 cases with weight loss > 10%, 72 controls)	GA ≥ 37 weeks, BW: ≥2500 g, exclusive breastfeeding, no congenital or infectious diseases	Formula feeding, medical conditions affecting feeding	Mean age: 3.4 ± 1.3 days	Serum sodium > 150 mEq/L in newborns with weight loss > 10%	13.9 ± 1.2%	NR	<149 mEq/L	Range 151–168 mEq/L
Boskabadi, 2010, Iran [5]	Matched 1:1 case–control study	106 term newborns (53 with dehydration, 53 without dehydration)	GA 37–40 weeks BW: 2500–4000 g; exclusive breastfeeding; no congenital or infectious diseases	Neonatal disease, formula feeding	Admission age: 3–10 days Median age: 6.2 days	Serum sodium ≥ 150 mEq/L with clinical dehydration and significant weight loss	16.2% ± 5.9%	1.6% ± 2.7%	137.8 mEq/L	160.1 mEq/L
Ergenekon, 2007, Turkey [21]	Prospective follow-up study	Term newborns with dehydration from a total of 2792 births	GA ≥ 37 weeks Mean BW: 3300 g, exclusive breastfeeding, no congenital or infectious conditions.	Systemic diseases, formula feeding, metabolic disorders	Admission age: 2–8 days	Serum sodium ≥ 150 mEq/L, significant weight loss (>10%) and clinical dehydration	11.5% ± 5.9%	NR	NR	156.4 ± 9.7 mEq/L
Bolat, 2013, Turkey [22]	Retrospective analytical study	81 term newborns of 4280 live births	GA ≥ 37 weeks, breastfed infants, no formula feeding, no major comorbidities	Congenital or metabolic disorders	Admission age: 3–11 days Mean age 6.2 days	Serum sodium ≥ 150 mEq/L, with weight loss > 10% and clinical signs of dehydration	17.9% ± 5.6%	NR	NR	158.1 ± 5.2 mEq/L
Oddie, 2001, United Kingdom [23]	Retrospective population-based study	32,015 live births; 907 readmissions evaluated	GA 37–41 weeks BW: 2350–4220 g, all firstborn, exclusively breastfed.	Not reported	Admission day: 6–10 days Median age: 7 days	Serum sodium ≥ 150 mEq/L, signs of dehydration corrected with fluids	Range 15–27%	NR	NR	Range 150–170 mEq/L
Miyoshi, 2020, Japan [24]	Retrospective cohort (observational)	Term newborns in a Baby-Friendly accredited hospital. Total N: 399 exclusively breastfed newborns included	GA ≥ 37 weeks	Multiple births, NICU admission, transfer, exclusive formula feeding	Admission age: 5–6 days	Operational definition based on weight loss ≥ 10%; serum sodium assessed only in subset.	9.4%	NR	NR	Range: 148–154 mEq/L
Carvalho, 2002, Portugal [25]	Retrospective hospital case study	12 dehydration cases among 4648 live births	Term newborns Mean BW: 3520 g Exclusive breastfeeding	Non-exclusively breastfed newborns	Admission age: 2–5 days	Clinical diagnosis, serum sodium ≥ 150 mEq/L	11.5%	NR	NR	153 mEq/L; (range 150–162 mEq/L)
Ozbek A., 2008, Turkey [26]	Case–control study	Cases 21, Controls 43	Term infants, ≤10 days old, exclusively breastfed, no feeding abnormalities, and healthy mothers without breastfeeding-related conditions.	No specifics reported	Mean age: 4.4 days	Weight loss ≥ 10%, dehydration signs, serum sodium > 150 mEq/L, elevated breast milk sodium.	11.3% ± 1.9%	NR	NR	152.2 ± 3.5 mEq/L
Celik et al., 2021, Turkey [27]	Prospective case–control study	43 term newborns (47 NHD cases, 96 healthy controls)	GA ≥ 37 weeks Mean BW: cases 3361 ± 379 g and controls 3219 ± 316 g, exclusively breastfed.	Newborns with systemic diseases or orofacial abnormalities; nipple/breast disorders preventing breastfeeding	Median age: 3 days	Serum sodium ≥ 150 mEq/L, weight loss ≥ 10%, and clinical signs of dehydration.	11.9% ± 3.2%	4.3% ± 2.5%	140 ± 2.6 mEq/L	155 ± 3.5 mEq/L

Abbreviations: NHD, neonatal hypernatremic dehydration; GA, gestational age; BW, birth weight; NICU, neonatal intensive care unit; NR, not reported. For sodium, mmol/L and mEq/L were standardized as equivalent units for reporting.

## Data Availability

No new data were created or analysed in this study.

## Supplementary Materials

The following supporting information can be downloaded at: https://www.mdpi.com/article/10.3390/jcm15134975/s1. Table S1: PRISMA2020 statement [36]; Table S2: Search Strategies; Table S3: Methodological Quality Assessment and Risk of Bias of Included Cohort Studies Using the Newcastle–Ottawa Scale; Table S4: Methodological Quality Assessment and Risk of Bias of Included Case-Control Studies Using the Newcastle–Ottawa Scale; Table S5: Methodological Quality Assessment and Risk of Bias of Included Cross-Sectional Studies Using the Joanna Briggs Institute tool.
